# Dual mobility acetabular component in revision total hip arthroplasty for persistent dislocation: no dislocations in 50 hips after 1–5 years

**DOI:** 10.1007/s10195-014-0318-7

**Published:** 2014-09-24

**Authors:** M. van Heumen, P. J. C. Heesterbeek, B. A. Swierstra, G. G. Van Hellemondt, J. H. M. Goosen

**Affiliations:** 1 Department of Orthopaedic Surgery, Sint Maartenskliniek, PO Box 9011, 6500 GM Nijmegen, The Netherlands; 2 Department of Research, Sint Maartenskliniek, Nijmegen, The Netherlands

**Keywords:** Revision hip arthroplasty, Dislocation, Dual mobility cup, Implant survival

## Abstract

**Background:**

A dual mobility cup has the theoretic potential to improve stability in primary total hip arthroplasty (THA) and mid-term cohort results are favorable. We hypothesized that use of a new-generation dual mobility cup in revision arthroplasty prevents dislocation in patients with a history of recurrent dislocation of the THA.

**Materials and methods:**

We performed a retrospective cohort study of patients receiving an isolated acetabular revision with a dual mobility cup for recurrent dislocation of the prosthesis with a minimum follow-up of 1 year. Kaplan–Meier survival analyses were performed with dislocation as a primary endpoint and re-revision for any reason as a secondary endpoint.

**Results:**

Forty-nine consecutive patients (50 hips) were included; none of the patients was lost to follow-up. The median follow-up was 29 months (range 12–66 months). Two patients died from unrelated causes. Survival after 56 months was 100 % based on dislocation and 93 % (95 % CI 79–98 %) based on re-revision for any reason. Radiologic analysis revealed no osteolysis or radiolucent lines around the acetabular component during the follow-up period.

**Conclusion:**

The dual mobility cup is an efficient solution for instability of THA with a favorable implant survival at 56 months.

**Level of evidence:**

Level 4, retrospective case series.

## Introduction

The risk of dislocation after total hip arthroplasty (THA) varies from 0.4−8.7 % for primary procedures and from 5−20 % for revisions [1]. Many patient and surgical risk factors for dislocation are described including female gender, older age at the time of surgery, previous hip surgery and revision surgery, neuromuscular disorders, poor medical status/high American Society for Anesthesiologists score (ASA score) and a small diameter of the femoral head [2–5].

**Fig. 1:**
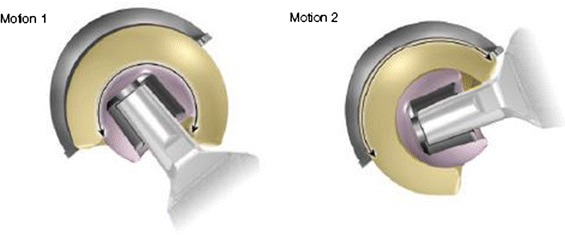
The biomechanical concept of the dual mobility cup consists of a double articulation—between femoral head and liner and between liner and cup. The first motion occurs between the small femoral head and the inside of the polyethylene liner, until the neck of the femoral stem comes into contact with the liner. The secondary motion occurs between the outside of the polyethylene liner and the metal acetabular cup, when a larger range of motion is required. Here the polyethylene liner acts as a large femoral head

On-going research has led to the development of many different improvements in the design and technique of the THA in an attempt to reduce the rate of dislocation. If no clear malposition of prosthetic components was present, large femoral heads, acetabular augmentation rings and constrained tripolar prostheses could be used. Although all have shown a reduction in dislocation rates, the results were still unsatisfactory [5–9]. Another development was the dual mobility cup which was devised by Dr. Bousquet in the mid-1970s [10]. The dual mobility cup is a combination of two fundamental principles—(1) the smaller the head articulating against a polyethylene liner, the lower the wear rates because of low friction [11], and (2) the larger the diameter of the bearing, the greater the joint stability [12] (Fig. 1).

The application of a dual mobility cup has been described for both primary and revision THA, as well as without a reason for persistent dislocation [16, 17, 19–22] and high risk of dislocation [13, 14]. Furthermore, there are only a few reports concerning the use of this type of implant in revision cases for recurrent dislocation. Leiber-Wackenheim et al. [15] reported on a group of 59 patients with a mean follow-up of 8 years. There was one early dislocation without recurrence and all implants survived. Hailer et al. [18] described a series of 228 cases with a follow-up of 2 years. They observed a survival of 99 % (95 % CI 97–100) based on dislocation and 93 % (95 % CI 90–97) based on the revision rate for any reason.

In order to test this theoretic advantage in stability of the THA, we investigated the dislocation rate of a dual mobility cup used for revision in 49 patients (50 hips) with a history of recurrent dislocation of their THA. We hypothesized that use of this component in revision arthroplasty would decrease the risk of re-dislocation of the THA. A second aim of the study was to assess the survival of the component.

## Materials and methods

**Fig. 2:**
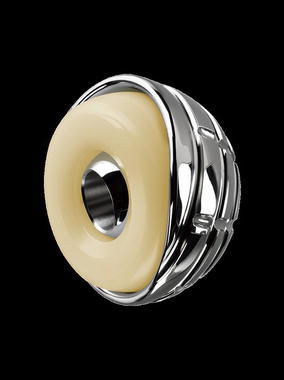
The cemented version of the dual mobility cup (Avantage^®^)

We performed a single-center retrospective study of patients who received an isolated acetabular revision with a dual mobility cup (Avantage^®^; Biomet, Warsaw, IN, USA) between January 2007 and June 2011. This cup has an uncemented shell design (coated with hydroxyapatite) or a polished shell for cementation (Fig. 2). The liner is made from argon-sterilized ultra-high molecular weight polyethylene (Arcom^®^; Biomet). Inclusion criteria were indication for revision with a dual mobility cup for recurrent dislocation or subluxation of the prosthesis (more than two episodes) and a minimum follow-up of 1 year after revision surgery. In total, 50 consecutive hips of 49 patients (one bilateral case) were included.

Surgery was performed using a posterolateral approach with the patient lying in a lateral decubitus position.

Postoperative management consisted of immediate full weight-bearing, using crutches for support, unless the intraoperative bone quality was poor and/or the surgeon used bone impaction grafting for reconstruction of the acetabulum. In these cases, partial weight-bearing over 3 months (15 % weight-bearing during the first 6 weeks, followed by 50 % for the next 6 weeks) was advised.

The clinical and radiologic data were retrieved from patient files. Demographic parameters included gender, age, height, weight, body mass index (BMI), ASA score, medical and surgical history, and side of planned surgery (Table 1). Primary indication and surgical history of the patients are presented in Tables 2 and 3. One patient with a history of seven surgeries prior to the revision had some traumatic dislocations of the hip prosthesis, requiring several open re-position revision surgeries. Another patient with a history of 11 surgeries prior to revision underwent several operations because of congenital hip dysplasia, followed by surgical lavage and a two-stage revision due to a joint infection of the primary THA, which was postoperatively complicated by persistent dislocation, leading to re-revision surgery. Thirty of the 50 cases had undergone two or more previous surgeries to the affected hip. In 23 cases, no previous revision surgery had been performed prior to the revision with the dual mobility cup; therefore, 27 of the procedures were re-revisions (Tables 4, 5). No additional pathologies with impact on the dislocation rate, like neurologic disorders, were found.

**Table 1:** Patient characteristics

Characteristics	*N*
Gender	
Male	10
Female	39
Mean height	170 cm (range 153–195 cm)
Mean weight	79 kg (range 40–120 kg)
Mean BMI	27.17 kg/m^2^ (range 16.6–43.0 kg/m^2^), with 34 patients overweight (BMI >25)
Mean age at operation	67 years (range 32–90 years)
Mean ASA-score	2.02 (range 1–3)

**Table 2:** Indication primary THA

Diagnosis	*N*	%
Osteoarthritis	31	62
Congenital hip dysplasia with secondary osteoarthritis	12	24
Medial collum fracture	3	6
Femoral head necrosis (after medial collum fracture/acetabular fracture with central luxation of the femoral head)	4	8

**Table 3:** Surgical history

No. of surgical procedures of the affected hip before revision with the dual mobility cup	No. of patients	%
1	20	40
2	14	28
3	6	12
4	5	10
5	3	6
6	0	0
7	1	2
8	0	0
9	0	0
10	0	0
11	1	2

**Table 4:** Revision surgery for any reason

No. of revisions for any reason, before revision with the dual mobility cup	No. of patients	%
0	23	46
1	17	34
2	4	8
3	4	8
4	2	4

**Table 5:** Revision surgery for instability

No. of revisions for instability, before revision with the dual mobility cup	No. of patients	%
0	29	58
1	17	34
2	2	4
3	2	4

Data regarding the type and size of implant, fixation method, technical details (Table 6) and complications, as well as information on any other occurring complications during the entire hospitalization, including infection, thrombosis, pulmonary embolism, hematoma, skin necrosis, nerve injury and/or death were obtained.

**Table 6:** Operative characteristics

Characteristic	*N*
Operated side	
Left	24
Right	26
Size of acetabular cup	
48	5
50	19
52	7
54	14
56	3
58	1
60	1
Femoral head size	
22	5
28	45
Fixation	
Cemented	46
Uncemented	4
Bone impaction grafting	
Yes	6
No	44

Postoperatively, outpatient clinic visits were routinely scheduled for radiologic (acetabular inclination angle and loosening of the cup) and clinical follow-up at 6 weeks, 3 months, and 1 year and were continued annually. Follow-up endpoints were dislocation of the THA, re-revision of the THA or death for any reason. Patients who did not attend the outpatient clinic visits for more than 1 year were contacted by telephone to ask for any dislocations or re-revisions postoperatively. When patients died, the general practitioner was contacted to obtain information on dislocations and implant re-revisions.

Descriptive statistics were presented as frequencies, and median values with ranges. Two Kaplan–Meier survival analyses were performed; one to estimate the cumulative probability of remaining free of dislocation, and the other to estimate the cumulative probability of remaining free of revision. The survival analysis was truncated when the number of patients remaining in the sample reached ten percent of the initial population. All statistical analyses were performed using STATA version 10.1 for Windows.

## Results

None of the 49 patients (50 hips) were lost to follow-up. Two patients died (of unrelated causes) before final analysis. The median time from revision surgery to evaluation was 29 months (range 12–66 months).

No postoperative dislocations were observed during follow-up. At final follow-up, three of the hips revised with a dual mobility cup had been re-revised. In one case, a two stage re-revision took place because of a postoperative joint infection 7 months after surgery. The second case was also a postoperative joint infection where the prosthesis was removed and left with a Girdlestone procedure. In the third case, there was a cup loosening based on an undersized uncemented shell (technical/surgical failure) directly after the revision and this was re-revised on the same day. In addition, three patients required re-operation with retention of the prosthesis—two of these patients required a wound revision, following debridement and early antibiotic treatment due to prolonged effusion of the wound. Tissue cultures showed a postoperative joint infection, which was managed by continuing antibiotic treatment for 3 months. During follow-up, the prosthesis could be retained and there were no signs of persistent infection. The third patient underwent re-operation due to sciatic nerve palsy. Drainage of a compressive hematoma was performed and the patient fully recovered after 4 months. Radiographic analysis revealed a mean acetabular inclination of 49° (range 31–65°). No osteolysis or radiolucent lines occurred around the acetabular component during the follow-up period.

**Fig. 3:**
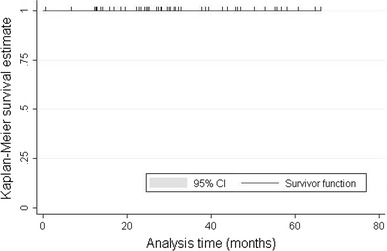
Cumulative survival of 50 prostheses with dislocation defined as failure event. The small vertical spikes represent censored data

**Fig. 4:**
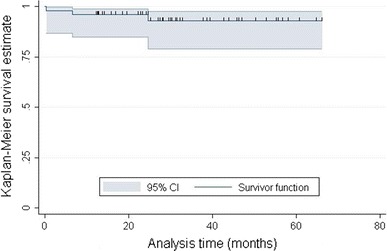
Cumulative survival of 50 prostheses with revision for any reason defined as failure event. The small vertical spikes represent censored data

The mean cumulative survival for remaining free of dislocation after 56 months was 100 % (Fig. 3). The mean cumulative survival for remaining free of revision for any reason after 56 months was 93 % (95 % CI 79–98) (Fig. 4).

## Discussion

Implant survival in our study (93 %) was comparable with other reports in the literature [13–20].

The current study population consisted of patients with an isolated acetabular revision due to recurrent dislocation of their THA. Most previous reports show comparable favorable results [13–18]. Langlais et al. [13] reviewed the results of 88 isolated acetabular revisions (82 patients at high risk of dislocation) using cemented dual mobility cups with a mean follow-up of 3 years (range 2–5 years). There was one dislocation (1.1 %) and survival was 94.6 % (two cases of aseptic loosening). Götze et al. [14] described their experience with an acetabular or total hip revision with a dual mobility cup (as used in our study) in 27 patients with a high risk of dislocation (14 cases) or a history of recurrent dislocation (13 cases). At a mean follow-up of one and a half years, there had been one dislocation of the polyethylene liner and the implant survival rate was 100 %. Leiber-Wackenheim et al. [15] are one of the few who reported on a series of isolated acetabular revisions with an uncemented dual mobility cup in a group of 59 patients with a history of recurrent dislocations. There was one early dislocation without recurrence after a mean follow-up of 8 years. All implants survived, and no component explantations were required. Civinini et al. [16] performed a prospective study of 33 patients (33 hips) with isolated acetabular revision with a dual mobility implant as used in the current study. Indication for revision was aseptic loosening (32 cases) or malposition of the cup (one case). At a mean follow-up of 3 years, no dislocations had occurred and survival rates were 97 % (95 % CI 82–98). Philippot et al. [17] showed the results of 163 acetabular revisions with a dual mobility cup. At a mean follow-up of 5 years, there were six cases (3.7 %) of dislocation and two cases of acetabular loosening; cup survival was 96.1 % (95 % CI 93–99). Recently, Hailer et al. [18] identified 228 THA cup revisions from the Swedish Hip Arthroplasty Register in patients with persistent dislocations with a dual mobility component as used in our study. They were only able to detect re-operations. At 2-year follow-up, they observed a survival of 99 % (95 % CI 97–100) based on dislocation and 93 % (95 % CI 90–97) based on the revision rate for any reason.

In contrast with the favorable results described above and the results found in the present study, Massin and Besnier [19] performed acetabular revisions using an uncemented dual mobility cup in 23 patients and reported a re-dislocation rate of 8.7 % at a mean follow-up of 4.5 years (range 2–10 years). Guyen et al. [20] reported on a series of 54 patients operated with a dual mobility cup at revision THA. At a mean follow-up of 3.9 years (range 2–6), the redislocation rate was 5.5 %.

In primary THA, survival rates after use of a dual mobility component were comparable [21–23] to the results of the present study. Philippot et al. [21] reported on a large series of 384 patients operated on with a dual mobility cup at primary THA. At a mean follow-up of 15 years (range 12–20), there were 14 cases (3.6 %) of dislocation (intraprosthetic dislocation: femoral head dislocates from liner) with an overall survival of 97 %. Bouchet et al. [22] performed a case–control study of primary THAs with use of a dual mobility cup in 105 patients, compared with the use of conventional implants in a matched group of 106 patients. At a mean follow-up of 4.3 years (range 3.2–5.6 years) there had been no dislocations in the dual mobility group versus five dislocations (4.6 % dislocation rate) in the matched group. Survival was 100 %. In a case series of ten THA patients with cerebral palsy no dislocations were observed at 39-month follow-up [23].

The main limitations of our study are the retrospective design and the lack of long-term follow-up (median 29 months; range 12–66 months). However, most dislocations occurred in the first 3 months postoperatively [24] and most re-revisions due to re-dislocation should have been performed during the first 2 years postoperatively [25]. We truncated the survival analysis at 56 months when only five patients remained.

Another limitation of the study is the absence of detailed functional results of the THA according to a clinical scale. These data would have provided more information about the functional performance of the implant. The study also included only a relatively small number of patients (49 patients, 50 hips). However, large series of isolated acetabular revisions concentrated on patients with recurrent dislocations are relatively uncommon in the literature. The strength of our study is the well-described homogeneous patient group. The results are comparable with the few other reports on this topic. This reinforces the favorable results of this type of implant in difficult revision cases.

In conclusion, the present study demonstrates an excellent 5-year survival rate with respect to the occurrence of postoperative dislocation with a dual mobility cup in revision THA due to instability. The re-revision rate for any reason is also promising. Thus, the dual mobility cup seems to be an efficient solution in revision cases for persistent dislocation of the THA. However, longer follow-up of a larger study population is required to confirm these relatively short-term findings and before firm conclusions can be drawn.
